# Advances in rare cell isolation: an optimization and evaluation study

**DOI:** 10.1186/s12967-016-1108-1

**Published:** 2017-01-05

**Authors:** Stefan Schreier, Piamsiri Sawaisorn, Rachanee Udomsangpetch, Wannapong Triampo

**Affiliations:** 1 Department of Physics, Faculty of Science, Mahidol University, 999 Phuttamonthon 4 Road, Salaya, 73170 Thailand; 2 Faculty of Medical Technology, Mahidol University, 999 Phuttamonthon 4 Road, Salaya, 73170 Thailand; 3 Centre of Excellence in Mathematics, CHE, 328 Si Ayutthaya Road, Bangkok, 10400 Thailand; 4 Thailand Center of Excellence in Physics, 328 Si Ayutthaya Road, Bangkok, 10400 Thailand

**Keywords:** Circulating tumor cells, Negative selection, CD45, Magnetic beads, Separation, Rare cells, Liquid biopsy

## Abstract

**Background:**

Rare nucleated CD45 negative cells in peripheral blood may be malignant such as circulating tumor cells. Untouched isolation thereof by depletion of normal is favored yet still technological challenging. We optimized and evaluated a novel magnetic bead-based negative selection approach for enhanced enrichment of rare peripheral blood nucleated CD45 negative cells and investigated the problem of rare cell contamination during phlebotomy.

**Methods:**

Firstly, the performance of the magnetic cell separation system was assessed using leukocytes and cultivated fibroblast cells in regard to depletion efficiency and the loss of cells of interest. Secondly, a negative selection assay was optimized for high performance, simplicity and cost efficiency. The negative selection assay consisted of; a RBC lysis step, two depletion cycles comprising direct magnetically labelling of leukocytes using anti-CD45 magnetic beads followed by magnetic capture of leukocytes using a duopole permanent magnet. Thirdly, assay evaluation was aligned to conditions of rare cell frequencies and comprised cell spike recovery, cell viability and proliferation, and CD45 negative cell detection. Additionally, the problem of CD45 negative cell contamination during phlebotomy was investigated.

**Results:**

The depletion factor and recovery of the negative selection assay measured at most 1600-fold and 96%, respectively, leaving at best 1.5 × 10^4^ leukocytes unseparated and took 35 min. The cell viability was negatively affected by chemical RBC lysis. Proliferation of 100 spiked ovarian cancer cells in culture measured 37% against a positive control. Healthy donor testing revealed findings of nucleated CD45 negative cells ranging from 1 to 22 cells /2.5 × 10^7^ leukocytes or 3.5 mL whole blood in 89% (23/26) of the samples.

**Conclusion:**

Our assay facilitates high performance at shortest assay time. The enrichment assay itself causes minor harm to cells and allows proliferation. Our findings suggest that rare cell contamination is unavoidable. An unexpected high variety of CD45 negative cells have been detected. It is hypothesized that a rare cell profile may translate into tumor marker independent screening.

## Background

Peripheral blood nucleated cells lacking the cell membrane receptor tyrosine phosphatase CD45 (CD45neg cells) are rare events and of interest in biomedical research. Focus of investigation has been stem and progenitor cells [1, 2], fetal and adult nucleated erythrocytes [3–5], CD45 deficient leukocytes [6, 7] and circulating cells of endothelial, epithelial or mesenchymal origin [8–10]. Elevated levels of CD45neg cells are abnormal to the peripheral blood cell spectrum and may represent pathological conditions such as autoimmunity, immune deficiency and hematologic, cardio-vascular or other malignancies [11–15]. In oncology, nucleated CD45neg cells of epithelial or mesenchymal type are in suspicion of being malignant in the setting of confirmed cancer cases, and are believed to have originated from a primary or secondary tumour, hence are referred to as circulating tumour cells (CTC) [14]. It is in particular the CD45neg CTC that has gained attraction as reliable diagnostic, predictive as well as prognostic marker and future CTC detection assays may be used for routine patient management [16, 17]. Recently, efforts to culture CTCs were purposed to investigate functional properties, pheno- and genotype, to test drug sensitivity as to support personalized therapy and/or to screen and develop new drugs [18, 19].

In general, investigations on CD45neg cells may require pre-enrichment at best selective enrichment. In oncology, numerous methods have been proposed for the enrichment of CTCs, yet still do not meet much needed quality [20–22]. Particular interest is amounting in assays capable of enriching exclusively CD45neg CTCs and is done by the depletion of at best all nucleated CD45 positive cells [23–25]. Abnormal cells are then made detectable amongst residual CD45 positive and normal CD45neg cells. The indirect isolation strategy carries no bias in phenotype or physical characteristics, yields untouched cells ideal for culture, allows multiple marker analysis and further downstream selection and gives rise to maximal yield of the cells of interest. The so called negative selection assays mostly employed immuno-magnetic bead separation technology [23, 26]. However, in praxis, low purities must be condoned due to relative high amounts of unseparated CD45 positive cells and a considerable loss of cells of interest [23, 27, 28]. Therefore, optimization may be required in demand of high performing assays. Few reports exist employing magnetic bead-based negative selection as sole enrichment step of CTCs and even fewer to focus on method optimization [26, 27, 29, 30]. Apparently, most optimization efforts aimed at the increment of leukocyte depletion by means of technological advancements in the magnetic capture technology. Commercial and in-house systems such as high gradient magnetic columns (Macs, PowerMag), quadrupole flow or microfluidic systems have been presented [27, 29, 30]. Meanwhile, a 1000-fold depletion of leukocytes is within range of possibility, yet must be considered as high. However, such solutions are complex when compared with the idea of simple magnetic separation in a plastic tube and in presence of a permanent magnet. Besides complexity and related disadvantages, previous capture systems require greater effort involving sterile preparations. Furthermore, assay quality criteria such as loss of cells of interest, the number of centrifugation steps, and assay time in general still have potential for improvements.

This study was primarily conducted to provide further advancement in rare cell enrichment technology with the following goals; (1) to selectively enrich nucleated CD45neg cells at high level based solely on magnetic bead technology, (2) to simplify the step of magnetic cell capture, (3) to reduce assay time as to reduce cell stress and foster cell preservation, (4) to minimize the loss in cells of interest and ultimately, (5) to benchmark the enrichment assay by detecting CD45neg cells from peripheral blood (PB). Technological advancement was made possible by a novel magnetic labelling method. The so called dynamic magnetic labelling method yields higher magnetically susceptible target cells (leukocytes) within shorter time when compared to mixing or keeping at rest, which then allows a relative simple magnetic capture system, reduces total assay time and expense of costly magnetic beads. This study presents a complete evaluation of the technical aspects of our negative selection assay including leukocyte depletion factor, cell recovery, cell viability as well as proliferative capability and the feasibility to enrich rare CD45neg cells from healthy donor PB. To detect any CD45neg cell, the enrichment assay was paired with two-colour fluorescence microscopy upholding the principle of negative selection based on unspecific cell staining by acridine orange fluorescent dye staining and CD45 positivity exclusion. We employed this largely unbiased isolation/detection approach to investigate the open question of false positive CTC identification by CD45neg cell contamination, which is believed to be incurred during phlebotomy.

To our knowledge, we are first to report negative selection assay enhancement by means of improvements on magnetic labelling. Major technical improvements have been achieved particularly in loss of cells of interest yielding near complete recovery of cell spikes and in enrichment assay time requiring 35 min. We detected a so far unreported diversity of CD45neg cells in healthy donor PB, which is considered to reflect the high selectivity of our isolation assay. Our findings did not support the idea of evading cell contamination such as epidermal cells by discarding the initial blood flow during phlebotomy. Moreover, this work has led us to the concept of pan-CD45neg cell profiling, which we believe could translate into tumour marker-independent early stage cancer detection.

## Methods

### Blood collection and processing

Blood samples were obtained from two sources: (1) buffy coats discarded from the routine blood bank processing at the Phramongkutklao Hospital, Bangkok, and (2) PB collected from volunteer healthy donors in both cases using standard 21G’ butterfly needle set. PB from healthy donors was taken by venous puncture collecting in total 12–14 mL in green-top BD Vacutainer blood collection tubes containing sodium heparin. Contamination by dermal cells during phlebotomy is currently assumed to occur and to be avoided by discharging the initial blood flow. To investigate the validity of the assumption, donations of fresh blood were further divided into a discharge sample comprising the initial 6 mL of blood flow and a so called clean sample containing the subsequent 6–8 mL of blood. The resulting three blood sample types; buffy coat, discharge and clean sample were investigated separately for content of CD45neg cells. Other than the evaluation of CD45neg cell content processing both PB discharge and clean samples at earliest 1 h and at latest 6 h after blood collection, PB was also used occasionally in optimization experiments as to compare results with buffy coat at most after 48 h and storage at room temperature. Buffy coats were stored at 4 °C and processed at most 48 h after collection. Buffy coats served in assay optimization experiments as source of leukocytes and in evaluation experiments as spiking medium. The study protocol was undertaken as approved by the institutional review board/independent ethics committee of Mahidol University. Informed consent was sought from blood donors at each time.

### RBC lysis

Standard chemical lysis buffer treatment was applied to remove red blood cells (RBC) (154 mM NH_4_Cl, 10 mM NaHCO_3_, 1 mM EDTA) adjusting the blood sample to lysis buffer ratio to 1:10 for buffy coat and 1:25 for PB. The cell suspension was incubated at 4 °C for a maximum of 5 min and subsequently centrifuged at 300×*g* for another 5 min. The cell pellet was resuspended in 10 mL PBS, supplemented with 0.5% bovine serum albumin and washed by centrifugation at 200×*g* for 10 min. The final cell pellet of nucleated cells containing contaminations of platelets and RBCs was resuspended in 100 µL Gibco® *Advanced RPMI* 1640 and kept at 4 °C until use. The cell numbers of nucleated cells were determined by hemocytometer (Neubauer) and subjected to experimentation within 1 h.

### Model CTCs

The cell lines L929 (fibroblasts derived from subcutaneous connective tissue) and A2780 (derived from ovarian cancer) served as model CD45neg cells (mCD45neg) and were cultured in DMEM medium (Gibco, USA) and RPMI-1640 medium (Gibco, USA), respectively. All cell cultures were supplemented with 10% FBS and 1% of Penicillin–Streptomycin (10,000 U/mL) (Gibco, USA) and incubated at 37 °C with 5% CO_2_ in a humidified atmosphere. Following culture, cells were harvested using 0.25% trypsin–EDTA into respective culture media and stored until use or for at most 7 days at 4 °C. Freshly harvested mCD45neg cells have been used in the experiments to assess cell viability using trypan blue (Sigma) dye staining.

### Assay performance evaluation

The overall assay performance was assessed by depletion factor, recovery, enrichment factor and magnetic bead efficiency. The depletion factor represents the ratio of CD45positive cells before depletion, denoted as L_total_ to the CD45 positive cell count after depletion, denoted as L_final_. The recovery of spiked cells represents the ratio of the initial spiked number of mCD45neg cells to the count of mCD45neg cells after depletion. The enrichment factor can be assessed in two ways. One is the mathematical product of depletion factor and recovery, another way goes by the ratio of purities of the mCD45neg cells before and after depletion [31]. The bead efficiency represented the amount of separable leukocytes per µL magnetic bead solution.

### Magnetic cell sorting

**Fig. 1:**
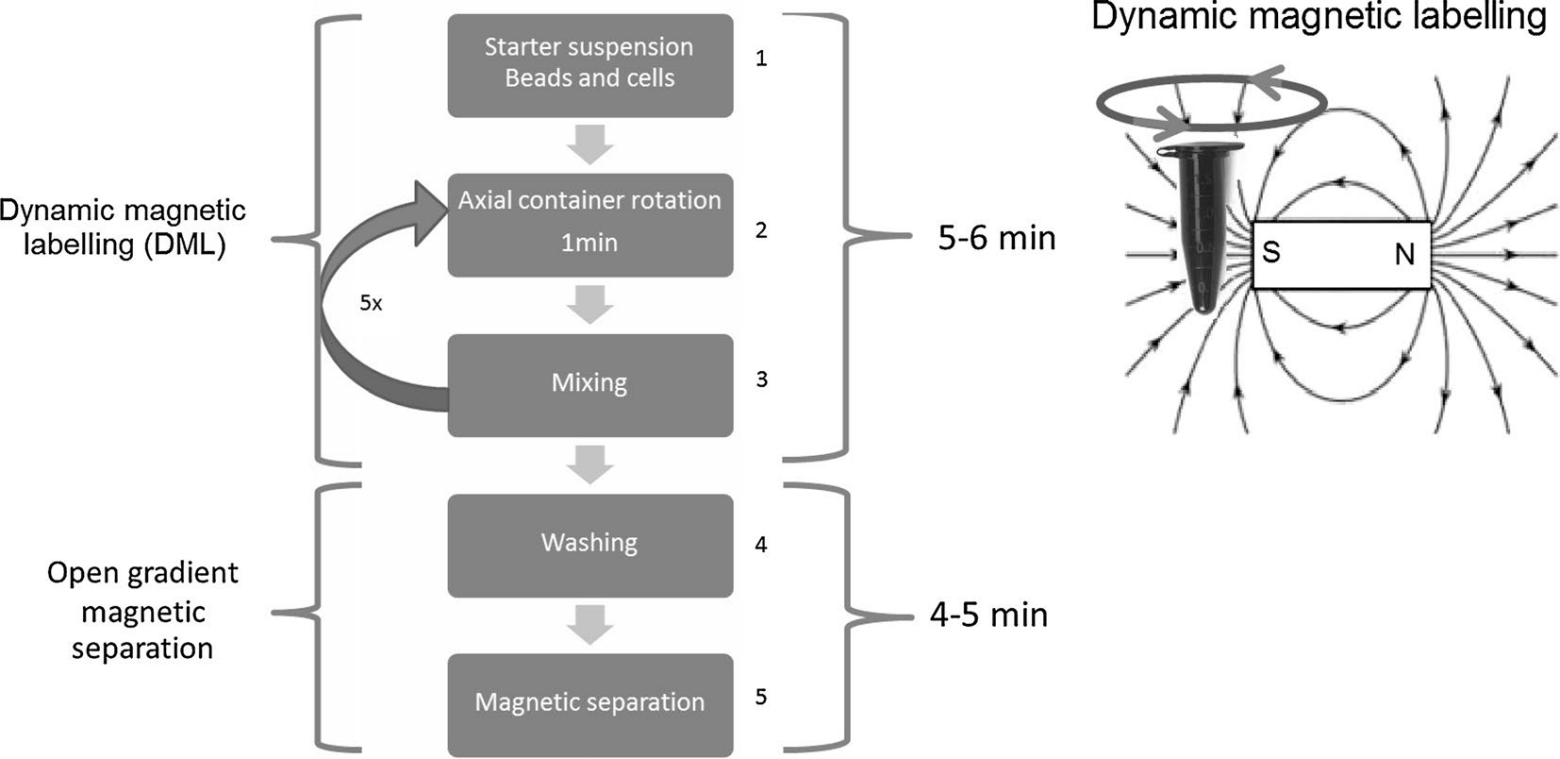
Summary of magnetic separation method. *Step 1* Mixing the beads with the cell suspension. *Step 2* Subjecting the incubation container to continued axial rotation at around 5–30 rpm in direct vicinity of a strong permanent magnet for 1 min. *Step 3* Mixing of the incubated suspension by continued pipetting and dispensing or vortexing. *Steps 2* and *3* have been repeated 5 times. *Step 4* Washing by diluting the incubated solution with cell-friendly buffer solution 1:1 and intensive mixing. *Step 5* Placing the incubation tube into the vicinity of a strong permanent magnet for magnetic capture and keep at rest for at least 4 min

The immuno-magnetic cell separation system comprised steps of magnetic labelling and magnetic capture. The magnetic labelling step was part of the negative selection assay improvement and was based on the method of dynamic magnetic labelling (Fig. 1).

Magnetic capture was facilitated by a duopole permanent magnet, which was constructed by pairing two equally squared neodymium magnets (387mT) in attracting configuration (overlapping of opposite poles) with a 5 mm air gap creating a high gradient magnetic field in the vicinity between and around the edges of both magnets. This site of highest magnetic effectiveness was in the air gap and used to place the incubation container after magnetic labelling. CD45 mouse anti-human IgG (clone MEM-28, Exbio, Praha) conjugated to magnetic nano-beads (Fluidmag-Ara, Chemicell, Berlin) have been used for direct magnetic labelling of leukocytes. The preparation of immune-magnetic beads employed the zero-length carbodiimide crosslinker method as previously described [32].

As part of the performance assessment and optimization procedure of the magnetic bead system, a standardized depletion experiment was set-up to determine the bead specificity and the efficiency of magnetic cell capture. The magnetic capture was translated into the bead efficiency. Hereby, 5 × 10^6^ leukocytes and 1 × 10^4^ fibroblast cells in pure solutions or mixed with leukocytes were incubated for 5 min in a 1.5 mL standard micro-centrifuge plastic tube with various volumes of magnetic beads and adjusted to 60 µL at room temperature. The total assay time was kept fix at 10 min and comprised 5 min of dynamic magnetic labelling, 1 min of washing and 4 min of magnetic capture. Subsequent to magnetic labelling, the incubated solution was mixed with 500 µL Gibco® *Advanced RPMI* 1640 and placed adjacent to the duopole permanent magnet at the location of the highest magnetic gradient. The depletion factor and bead efficiency was assessed by the leukocyte count in suspension after magnetic separation. The experiment outcome was the depletion factor and cell loss due to non-specific binding to magnetic beads.

As part of the assay evaluation, we isolated and detected CD45neg cells from healthy donor PB by negative selection and fluorescence microscopy. The complete CD45neg cell isolation/detection assay comprised coarsely the following steps; RBC-lysis, first centrifugation/cell pelleting step, second centrifugation/washing step, first depletion cycle, third centrifugation/cell pelleting step, second depletion cycle, fourth centrifugation/cell pelleting step, fluorescence marker labelling, fifth centrifugation/washing step and finally microscopic analysis. In detail, nucleated cell counts were adjusted to 2.5 × 10^7^ nucleated cells after RBC lysis from initially 6 mL whole blood or 1.5 mL buffy coat. The cell suspension during the first depletion cycle was adjusted to 250 µL including 75–80 µL beads. Magnetic labelling and capture was performed in the same manner as for the standard depletion experiments however, having increased the washing volume to 1 mL. Gibco® *Advanced RPMI* 1640 was used for incubation, washing, pelleting and resuspension of cells. After the first depletion cycle, uncaptured cell material was pelleted at 300×*g* for 5 min and at room temperature. The resulting smaller pellet was resuspended in 40 µL using 20 µL of magnetic bead solution and a second depletion cycle followed.

### Flow cytometry

Flow analysis was conducted on a flow cytometer BD FACSCanto II (BD Biosciences, San Jose, CA, USA). Cells suspensions contained at most 5 × 10^4^ cells and were stained with anti-CD45-PE (ebioscience) at 4 °C in 40 µL Gibco® *Advanced RPMI* 1640 for at least 20 min. Cell suspensions consisted of leukocytes before and after depletion treatment being the negative control, fibroblasts as positive control, and spiked samples before and after depletion.

### Microscopy analysis

Optimization experiments related to depletion efficiency employed exclusively leukocytes as obtained after RBC lysis. For microscopic analysis, a simple and fast method was chosen as to determine cell counts using acridine orange (AO) staining. In brief, 1 µL of AO stock solution (100 µg/mL) was added to various cell sample volumes ranging from 40 µL to 1 mL. In optimization experiments related to cell recovery, mCD45neg cells were stained prior to spiking in either buffy coats or leukocyte suspensions using CSFE following the manufacturer’s protocol without modifications (CFSE Cell Division Tracker Kit, Biolegend). Samples with either AO or CSFE fluorescence were loaded on hemocytometer (Neubauer) and analyzed using a Carl Zeiss Axioscope 2 fluorescence microscope equipped with a FITC filter set.

In assay evaluation experiments, CD45neg cells from buffy coat, PB discharge and clean samples after RBC lysis and leukocyte depletion were detected by two color fluorescence microscopy staining cells using AO fluorescence dye and anti-CD45PE conjugated antibodies (ebioscience). In brief, 30–40 µL of cell suspension was incubated with 3 µL anti-CD45 conjugate solution at 4 °C in the dark for at least 20 min followed by AO staining as already described for optimization experiments and then washed by centrifugation at 300×*g* and 5 min with 1.5 mL Gibco® *Advanced RPMI* 1640 and resuspended in 30 µL. The cell suspension was then loaded dropwise at 10–15 µL onto a microscopy slide without cover. Visualization of cell fluorescence was performed using a Nikon Eclipse TE2000-E inverted fluorescence microscope equipped with filter sets for fluorescent dyes FITC, phosphatidylethanolamine (PE) and Texas Red. Microscopic reading was done manual by visualization at 200× and 400× magnification spotting CD45neg cells under fluorescence green light emission and recording of suspicious cells using a Carl Zeiss Axiocam MR colour and MRGrab software under brightfield illumination as well as fluorescent light. CD45neg cells were identified as near round objects in sizes between 4 and 25 µm in diameter with clear contrast to background under brightfield visualization and appearing in AO fluorescence yet lacking PE fluorescence when measured against background intensity. AO fluorescence leaking into the PE channel slightly above background intensity was identified by equal fluorescence intensity distribution across the cell or higher intensity in the center of the cells as opposed to the ring-like fluorescence appearance of CD45PE positive cells throughout the emission light spectrum from 520 till 650 nm.

### Cell recovery

The recovery rate of the isolation assay was determined by spiking 2, 5 and 8 CFSE stained mCD45neg cells into buffy coat at volumes that corresponded to 2.5 × 10^7^ nucleated cells. Cells for spiking were selected from stock solutions by slowly drawing individual cells into a 10 µL plastic tip under microscopic vision at 100× magnification and fluorescence excitation using an inverted fluorescence microscope (Nikon Eclipse TE2000-E) equipped with FITC filter set. In brief, CSFE stained mCD45neg cells were diluted from stock (approx. 1 × 10^5^ cells/µL) to a final concentration of max. 5 cells/µL in one flat bottom well of a 96-well plate. The tip of a 10 µL pipette was introduced into the well bottom. The controlled and slow passage of strong green fluorescent cells into the pipette tip was counted and visualized by adjusting the focus layer to the tip end position. After the aspired cell number was reached, the tip was removed from the well and the cells within the pipette tip were expelled directly into the blood sample. The spiked sample was then subjected to RBC lysis, magnetic labelling and capture followed by microscopic analysis as described in “Magnetic cell sorting” section for negative selection of CD45neg cells.

### Cell viability and proliferative capability

Pure suspensions of freshly harvested mCD45neg were adjusted to contain 5 × 10^5^ cells and subjected to the exact same procedure of the negative selection assay including RBC lysis as described for buffy coat samples (“Cell recovery” section). Trypan Blue dye staining was used to determine viability of the harvested cells before the process, after RBC lysis, after the first depletion cycle and after the second depletion cycle.

Cell proliferation was assessed by spiking 100 freshly harvested ovarian cancer cells into buffy coat containing 2.5 × 10^7^ leukocytes. The spiked number of cells was determined using a hemocytometer (Neubauer). The same negative selection procedure was applied to the sample as described above yet requiring sterile preparations therefore working under laminar flow conditions and with sterile solutions. The resulting cell suspension after magnetic treatment was then diluted in 7.5 mL growth medium and transferred into 3 wells of a 6-well plate at equal volumes for culture. A positive control without any treatment was prepared containing the same starter amount of ovarian cancer cells and was subjected to culture under same conditions as the spiking sample. The growth medium remained unchanged until day 7. The cell proliferation in culture of the sample and the positive control were analyzed by an inverted brightfield microscope. Proliferative activity was defined as at least two clearly dividing, bottom attached cells and being significant larger than any nucleated CD45 positive cell.

### Statistics

One tailed t test and correlation analysis (Microsoft Excel) was employed for statistical analyses of the enriched CD45neg cell counts. p < 0.05 was considered statistically significant.

## Results

### Theoretical considerations

The optimization of immuno-magnetic separation system was guided by theoretical considerations about attainable assay performance. Scarcity of CD45neg cells in particular CTCs, translates into relative large sampling volumes and the requirement of a low limit of detection (LOD) measuring one cell per 1 to max. 10 mL blood [17]. One major goal of optimization was to reduce costs hence, bead expense. The predetermination of the lowest possible L_total_ may appear reasonable in view of a relative high cell-to-bead ratio and the high number of target cells. Given an assumed CD45neg cell frequency of 1 cell per 3 mL PB, a recovery of at least 80 and a 90% sensitivity of analysis, we may require at least 4.2 mL PB or a minimal L_total_ of 2.5 × 10^7^ cells (average 6 × 10^6^/mL) to be able to detect one cell of interest. Following this initial estimation, we identified and standardized our assay to the minimal L_total_ of 2.5 × 10^7^ leukocytes regardless the blood volume and aimed at a recovery that would support an assay LOD of 0.24 cells/mL.

Another performance criterion is the enrichment factor, which is presented as the mathematical product of cell recovery and the depletion factor or the ratio between purities of cells of interest before and after enrichment [31]. Both primary measures of performance, the depletion factor and the recovery usually stand opposed to each other in magnetic bead separation technology, as key is the amount of beads per sample as well as the rate of non-specific binding. Depletion factors were qualified according to standard performance in the field ranging till 14.5 as subnormal, till 33.5 as normal, till 100 as high, and depletion factors beyond 100 as ultra-high depletion. Targeting a recovery of 80% may seem reasonable when compared with other works [26]. We considered L_final_ counting 2–4 × 10^4^ leukocytes as tolerable interference during microscopic reading therefore, requiring at least 625-fold depletion for L_total_ equal to 2.5 × 10^7^ cells and 500-fold enrichment.

The ease and speed of sample processing, but also analysis would affect the sample throughput and costs per test. Shortened assay time may also reduce cell stress thus, help to reduce cell loss and as such improve the LOD and likelihood of cell proliferation in culture. It is declared goal to limit the assay time including analysis to 2 h. Key to speed is the dynamic magnetic labelling procedure. The performance parameters and their aspired values are listed in Table 1.

**Table 1:** Assay performance parameters

Parameter	Optimization goal	Definition	Dependencies
Limit of detection (LOD)	0.24 cells/mL peripheral blood	Detection of at least one cell of interest in 4.2 mL blood or 2.5e7 total cells	Test sensitivity, recovery, L_total_
Enrichment factor	>500	Ratio between the cell-of-interest frequency before and after enrichment	Depletion factor and recovery
Depletion factor	>625	Ratio between the leukocyte amount before and after enrichment	Bead amount high
Recovery	80%	Measure of target cell loss	Bead amount low
Initial leukocyte amount, L_total_	2.5 × 10^7^	Assay start with leukocyte cell count	Blood volume 3–7 mL, enrichment factor, cell of interest frequency,
Residual leukocytes, L_final_	2 × 10^4^ to 4 × 10^4^ cells	Leftover leukocytes after depletion	Depletion factor
Assay time	2 h	Complete assay time including isolation and analysis	RBC lysis quality and enrichment factor

### Magnetic separation system efficiency

**Fig. 2:**
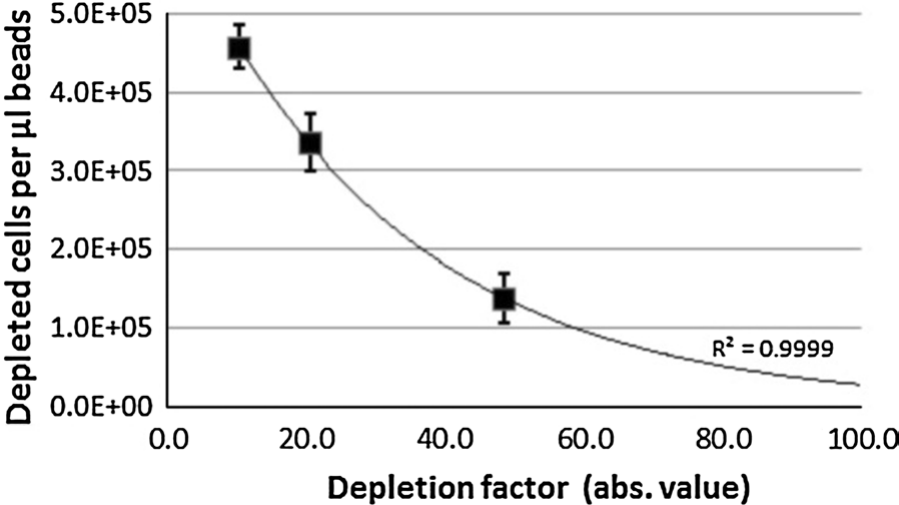
Bead efficiency. Quantitative assessment of the potential of the bead solution to separate targeted cells. The bead efficiency (±SD) was assessed in range of ×10 to ×50 depletion and measured 1.4 × 10^5^ to 4.6 × 10^5^ cells/µL. The trend line was assumed exponential

We then investigated the actual performance of the immuno-magnetic separation system in regard to depletion factor and cell loss carrying out time fixed standardized depletion experiments. We introduced bead efficiency as an indicator of depletion performance that we defined as the amount of magnetically separable cells/µL magnetic bead solution. This performance value helped us to predetermine the bead amounts required for attainable depletion levels at given cell amounts. The bead efficiency is influenced by the entire magnetic separation system that includes the magnetic bead reactivity, the magnetic labelling method, the magnetic capture technology and even the functional properties of the biological system. Magnetic bead volumes were varied using up to 50 µL then in principle adding cells to the magnetic bead solution at the given yet arbitrary maximal incubation volume of 60 µL. Figure 2 shows the bead efficiency as a function of depletion factor and reveals that the magnetic separation system was able to yield a high-level depletion factor within 10 min.

It was wishful to be in control of the depletion level for the later design and optimization of the negative selection assay. We understand from the results firstly, that the depletion factor can be predetermined by adjusting the right amount of magnetic beads and secondly, that depleting at higher depletion levels requires significant more bead expense. For subnormal depletion, 1 µL of bead solution was enough to separate around 4.8 × 10^5^ leukocytes. Yet, high-level depletion required around 3 times more bead volume to capture the same leukocyte amount using up almost all of the given incubation volume for the bead solution. A third observation was that the drop in bead efficiency with higher depletion factors did not follow a linear trend suggesting the elevation in bead efficiency with respect to the depletion level. As a matter of fact, the bead concentration increased as a consequence of lesser dilution of the bead stock solution. Higher bead concentrations were shown to enhance magnetic labelling and consequently magnetic capture [33]. The highest depletion level measured 65-fold depletion for this specific experiment set-up.

Besides the magnetic bead amount, the storage time of the blood sample was found to be of great influence on the system, in particular on the high depletion range. We tested whole blood samples aged 5, 24 and 48 h aiming at normal depletion and observed a significant reduction in bead efficiency measuring depletion factors of 25, 14.5 and 6.7, respectively. We reasoned that dead or apoptotic cells would be less separable and that its amount would increase with blood storage time.

The cell loss due to non-specific binding of the magnetic beads to the cells of interest was assessed at standard protocol conditions using 1 × 10^4^ fibroblast cells as pure suspension and spiked in leukocyte suspension (Table 2). The degree of non-specific binding is believed to depend amongst other factors on amount and concentration of magnetic beads and therefore, is expected to increase when seeking higher depletion factors. We also expected higher non-specific binding in pure fibroblast suspensions due to the significant higher bead to fibroblast cell ratio when compared to mixed cell suspensions. The supernatant after depletion was investigated for remaining fibroblast content and recorded in percent based on the initial spiked count of fibroblast cells. We observed substantial non-specific capture of fibroblast cells only in pure cell suspension and at the high depletion level. Nevertheless, in mixed suspension, the fibroblast loss at the high depletion level was notable with around 8%. The overall performance assessment of the cell separation system suggested that up to 98.4% leukocytes could be depleted without marked loss of cells of interest yielding a possible 58.2-fold enrichment factor within 10 min.

**Table 2:** Non-specific binding analysis

Depletion level	Cell loss in pure suspension (1 × 10^4^ fibroblasts) (%)	Cell loss in mixed suspension with leukocytes (1 × 10^4^ fibroblasts/5 × 10^6^ leukocytes) (%)
Subnormal (n = 3)	8.9 ± 0.8	0.0
Normal (n = 4)	13.4 ± 1.1	1.6 ± 1.1
High (n = 3)	36.3 ± 4.3	8.3 ± 2.3

### Negative selection assay

The results of the performance assessment in the previous section have been used to design a negative selection assay. Depleting leukocytes at the ultra-high level was hardly possible, and if so at excessive expense of costly beads. Therefore, for larger blood sample volumes, the elevation of the depletion factor by means of bead amount did not seem practical. One observation during performance assessment experiments was that a considerable minority of nucleated cells (data not shown) would not be easily separable, hence discriminating leukocytes into high and low reactive against the magnetic beads. Determined to reduce the expense of beads, the idea may seem plausible to employ two depletion cycles both comprising magnetic labelling and capture that would initially deplete the majority of highly reacting leukocytes yielding highest possible bead efficiency followed by the depletion of the fewer left-over and less reacting leukocytes however, at considerable lower bead efficiency. Based on the records of bead efficiency (Fig. 2), we intended to establish and test a repeated depletion protocol for the negative selection assay as shown in Table 3.

**Table 3:** Projection of attainable negative selection assay depletion performance

Incubation	*Ltotal*	Depletion factor	*Lfinal*	Bead efficiency (cells/µL)
First depletion	2.5 × 10^7^	20×	1.25 × 10^6^	3.4 × 10^5^
Second depletion	1.25 × 10^6^	50×	2.5 × 10^4^	1.4 × 10^5^
Total depletion	2.5 × 10^7^	1000×	2.5 × 10^4^	3.0 × 10^5^

**Fig. 3:**
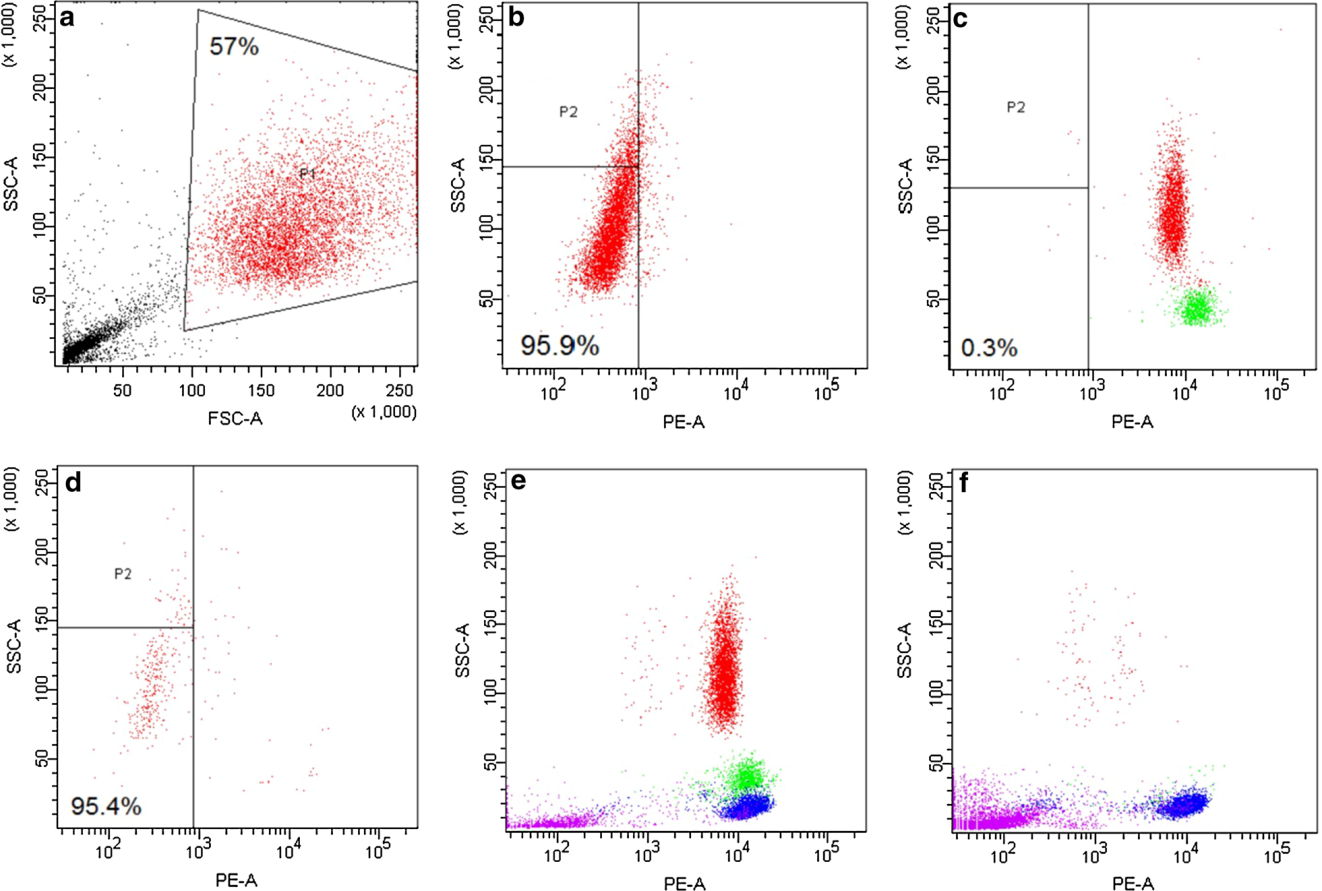
Flow cytometry of fibroblast spiking experiment. **a** Gated CD45PE stained fibroblast cells in *red*. **b** Plot of CD45PE stained fibroblast subpopulation gated by fluorescence mode. **c** Purified leukocytes solution containing 2.5 × 10^7^ normal cells and 1 × 10^4^ fibroblast cells before depletion as gated in **a**. The subpopulation in *red* comprises granulocytes. The population in *green* comprises monocytes. **d** Sample after depletion as gated in **a**. The *red* population comprises mainly fibroblasts. **e** Unspiked purified leukocyte suspension gated for all cell populations before depletion. The *red* population comprises granulocytes (35%), the *green* population comprises monocytes (10.9%), the *blue* population comprises lymphocytes (32.9%) and the *magenta* population comprises debris (13.5%). **f** Enriched leukocyte population. The *red* population comprises granulocytes (1.1%), the *green* population comprises monocytes (0.9%), the *blue* population comprises lymphocytes (26.6%) and the *magenta* population comprises debris (50.4%)

Our previous results showed decreased bead efficiency in dependence of blood age (“Magnetic separation system efficiency” section). Therefore, buffy coats as well as fresh blood samples were used before the resting time of 24 and 12 h, respectively. As part of the optimization process, cell losses along the process steps have been identified by spiking experiments using 1 × 10^4^ fibroblast cells and purified leukocyte suspensions adjusted to 2.5 × 10^7^ cells. The cell mixture was incubated with 75–80 µL of magnetic bead solution in 250 µL incubation volume during the first depletion step and with 20 µL bead suspension in maximal 40 µL during the second depletion step. Different incubation volumes between the two depletion cycles have been chosen to sustain a bead concentration that would facilitate fast bead-cell binding kinetics. In numerous repeats, the final depletion factor and the fibroblast recovery measured on average 888-fold (±395) and 92.8% (±3.3%), respectively and 1667-fold and 96.4% at best, resulting in a possible average 824-fold enrichment. The bead efficiency for the 2-step procedure using fresh blood not older than 12 h measured 2.63 × 10^5^/µL using maximal 95 µL of bead solution. The assay time not including analysis measured 35 min. The performance of the negative selection assay was qualitatively investigated by flow cytometry. Figure 3a–d depict the enrichment when gated specifically for the fibroblast fraction. Fibroblasts stained negative for CD45PE as expected (Fig. 3b). The final purity of the enriched fibroblast cells measured 95.4% from originally 0.3% when initially gating the fibroblast population. We also observed cell selective depletion efficiency (Fig. 3e, f). A near complete depletion of the monocyte and granulocyte fraction was achieved leaving a marked residual leukocyte amount in the lymphocyte fraction. This was in concordance with microscopy analysis showing that the majority of left-over leukocytes are relative round and small cell as can be seen in Figs. 5 and 6. Large cells formed the minority counting only 2200 cells on average in a suspension of 1.5–4 ×10^4^ remaining leukocytes.

### Cell recovery

Having adjusted a relative high cell spike in the preceding experiments for reasons of cell loss identification and qualitatively analysis by flow cytometry, spiking quantities that simulate true rare cell frequencies may give a closer estimate on the possible LOD of the isolation/detection assay. Therefore, we determined the recovery of ovarian cancer and fibroblast cells spiking 2, 5 and 8 cells into buffy coat. The leukocyte concentration in buffy coat was determined prior to spiking and the volume adjusted to contain 2.5 × 10^7^ leukocytes. Each spiking level has been repeated three times (Table 4). All experiments showed recovery of most of the cells. The two cell spikes were positive for both cell lines in all cases. Therefore, the assay may support a LOD of 0.33 cells/mL detecting at least 1 CD45neg cell in 1.25 × 10^7^ total cells or 3 mL fresh blood.

**Table 4:** Recovery of single cell spiking experiments

Spiked cells	Recovered ovarian cells	Recovered fibroblast cells	Average recovery (range)
8 (n = 3)	7, 5	5	70.8% (62.5–87.5%)
5 (n = 3)	4, 4	4	80% (80%)
2 (n = 3)	1,1	2	66.7% (50–100%)

### Cell viability and proliferation

**Fig. 4:**
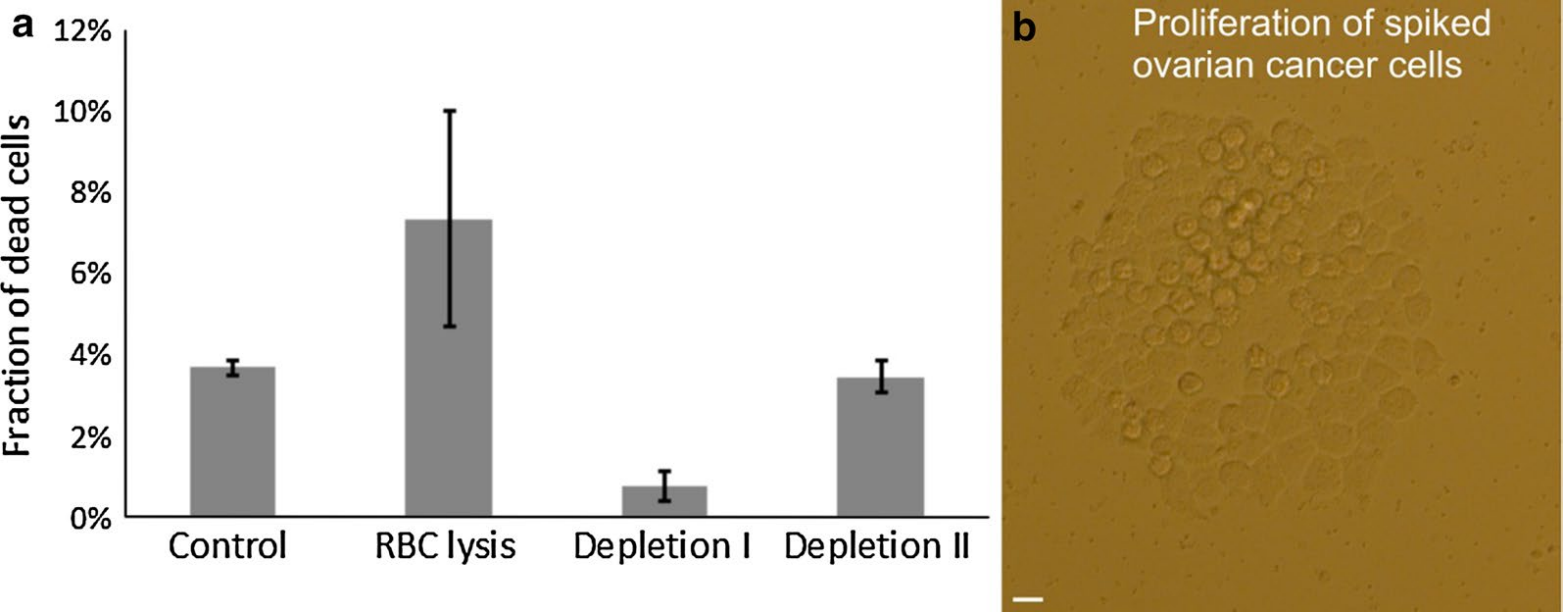
Cell viability and proliferation. **a** Viability assessment (±SD) of mCD45neg cells by Trypan Blue staining after each step of the negative selection assay. **b** Ovarian cancer cell outgrowth developing from 100 cell spike in buffy coat after negative selection procedure and 7 day culture. The *bar* represents the length of 20 µm

The viability or cell damage due to RBC lysis and the leukocyte depletion procedure was determined by trypan blue dye exclusion using ovarian cancer and fibroblast cell lines (Fig. 4a). The assay evaluation experiments were carried out once using fibroblasts and twice using ovarian cancer cells. The results were consistent between the two cell lines and suggested a slight decrease in viability according to one depletion cycle by maximal 3%. A relative high decrease in viability of maximal 9% was measured after chemical lysis of RBCs. The proliferative activity of 100 ovarian cancer cells was tested on a control and a cancer cell spiked blood sample. Dividing cells or cell outgrowth (Fig. 4b) was counted as a single event. The control sample counted 27 such events. The ovarian cells undergoing the enrichment treatment proliferated less when compared with the positive control, counting 10 events. The cell cultures were found to be free of bacterial contamination throughout the period of culture.”

### Isolation/detection assay feasibility

**Fig. 5:**
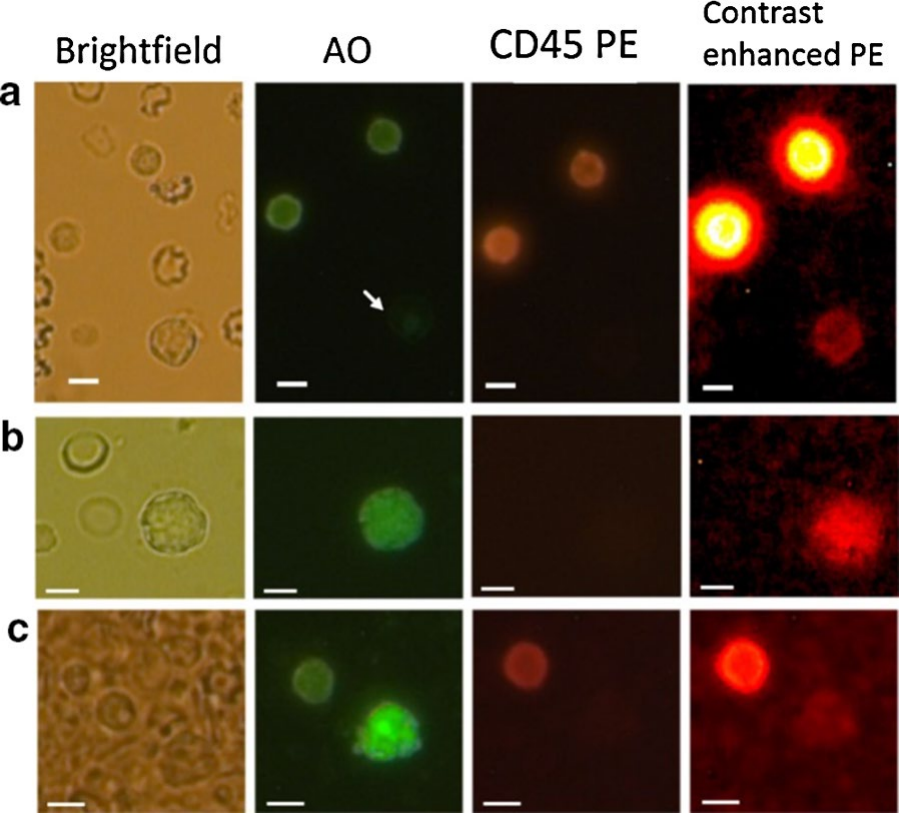
Cell image analysis. Cells are recorded at 4 different appearances from *left* to *right*; under brightfield, AO fluorescence, PE fluorescence and contrast enhanced (ImageJ manual brightness adjustment) PE fluorescence. The *bars* represent a length of 10 µm. **a** CD45 positive cell identification. The cell indicated by a *white arrow* shows low AO as well as *ring shaped* PE fluorescence. CD45 negative cell identification with normal (**b**) and high (c) AO fluorescence intensity without ring formation in the light wavelength above 550 nm. Residual fluorescence is pronounced (**b**) and at noise level (**c**), visible only after contrast enhancement. The fluorescence signal is ascribed to AO fluorescence only

Encouraged by the high cell recovery for lowest spiking numbers and the high cell viability, we went on to investigate the potential of our isolation assay to enrich CD45neg cells from PB. Moreover, we applied the assay to contribute to the ongoing debate about false positive CTC identification. Since there has not been identified any marker, which is solely characteristic to CTCs so far, we can presume that the most conserved CTC selection criteria are CD45 negativity and cell nucleation in size greater 4 µm. In order to serve both purposes, the feasibility assessment of the enrichment assay and the investigation of CTC false positives, we used two-color fluorescence microscopy for analysis based on cell nucleation and CD45 positivity exclusion sampling buffy coat, discharge and clean whole blood samples. Reader accuracy and linearity was assessed in side experiments using cell spikes across the range of 2–2000 mCD45neg cells in suspensions of 2 × 10^4^ leukocytes and followed by fluorescent dye staining confirming highest accuracy and linearity (data not shown). Naturally, most cells after enrichment stained positive for CD45 at varying fluorescence intensities and appeared typically as a ring (Fig. 5a). Consequently, true CD45neg cells did not show any fluorescence ring formation and appeared purely in AO fluorescence (Fig. 5b, c).

**Fig. 6:**
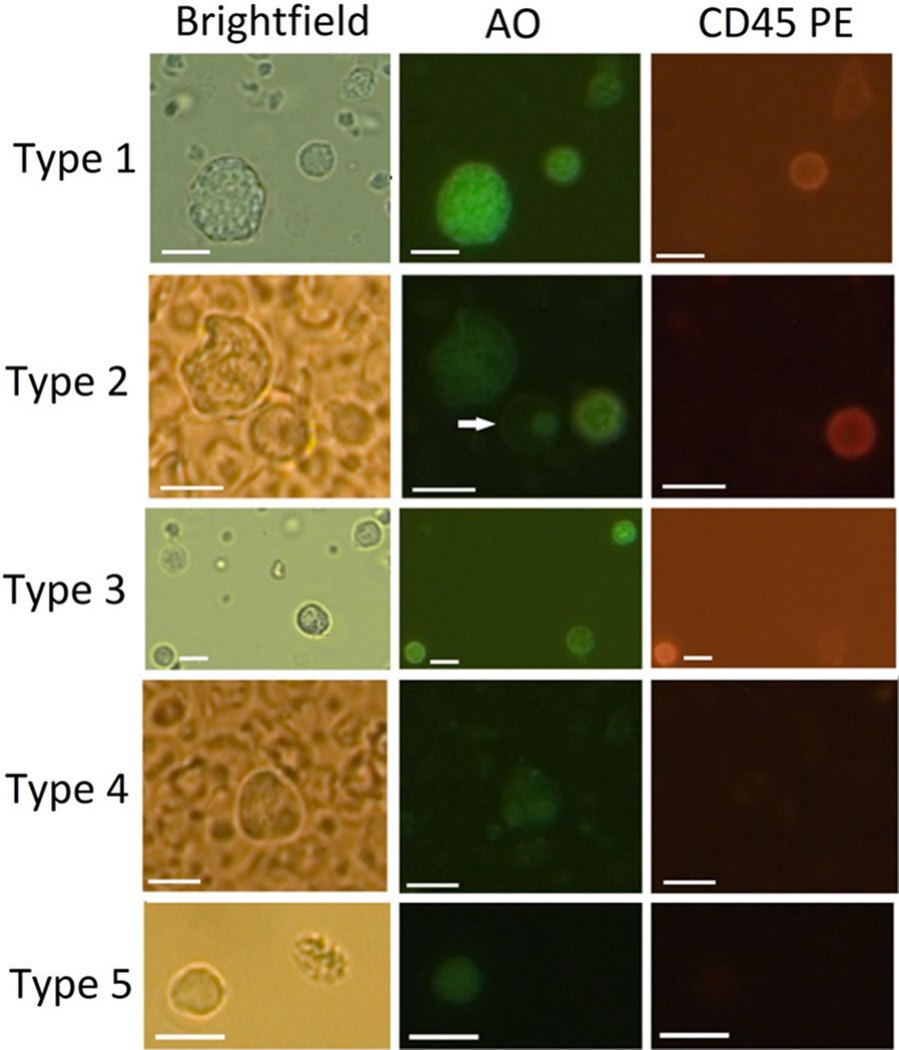
Images of CD45negative cells. Representative images of cells from different donors shown under brightfield, fluorescence green light and fluorescence red light. *Type 1* Large round/oval cell with strong AO fluorescence. *Type 2* Large irregular shaped cell with medium AO fluorescence. *Type 3* Medium sized round cell with two distinct high AO fluorescence patches. *Type 4* Medium-sized oval shaped cell with two distinct very low AO fluorescence nuclei. *Type 5* Small cell with equal medium AO fluorescence throughout the cell. The *bars* represent a length of 10 µm

We detected CD45neg cells in 88.9% of the sampled blood at frequencies from 1 to 22 cells (average 5.6 cells/2.5 × 10^7^ WBCs and in 58% (13/26) of the sampled blood exceeding 3 cells and including all three sample types (Table 5). Furthermore, the probability to recovery zero cells measured 11.5%. It shall be reminded that L_total_ counting 2.5 × 10^7^ leukocytes was crucial to the sampling. The average cell concentration in whole blood donors measured 7.0 × 10^6^ ± 1.5 × 10^6^ cells hence, sampling on average a blood volume of 3.6 mL. The CD45neg cells of interest greatly varied in size, morphology and AO fluorescence color and intensity. For the prevailing idea that cell abnormality in regard to tumor growth may be cell size dependent, we grouped the detected cells into three size categories. The sizing was referenced to the average diameter of normal nucleated CD45 positive cells in the depleted sample found to measure 8.4 ± 1.1 µm. Representative images of CD45neg cells at different sizes are shown in Fig. 6.

**Table 5:** CD45neg cell analysis in healthy blood samples

Sample type	Sample Nr.	Large cells (>10 µm)	Medium cells (7–10 µm)	Small cells (4 > 6 µm)	Sum of cells	Blood resting time (h)
Buffy coat (n = 9)	1	2	1	0	3	24
	2	1	0	0	1	
	3	2	2	0	4	
	4	1	0	0	1	
	5	2	4	0	6	
	6	0	0	0	0	
	7	4	4	2	10	
	8	1	1	0	2	
	9	0	0	0	0	
	Average	1.4	1.3	0.2	3	
Whole blood sample: clean collection after 6 mL (n = 9)	Donor 1	0	0	3	3	6
	Donor 2^a^	0	0	16	16	6
	Donor 3	1	2	0	3	6
	Donor 4	1	1	2	4	6
	Donor 5	0	4	2	6	6
	Donor 6^b^	12	1	1	14	3
	Donor 7	0	1	0	1	6
	Donor 8	3	3	0	6	3
	Donor 9^c^	6	9	7	22	1
	Average	2.6	2.3	3.44	8.3	
Whole blood sample: discharge collection of the first 6 mL (n = 8)	Donor 1	0	0	0	0	3
	Donor 2^a^	1	1	8	10	3
	Donor 3	3	1	0	4	3
	Donor 4	1	1	3	5	3
	Donor 5	N.A.	N.A.	N.A.	N.A.	3
	Donor 6^b^	4	0	0	4	6
	Donor 7	1	1	0	2	3
	Donor 8	3	2	0	5	6
	Donor 9^c^	7	8	2	17	1
	Average	2.5	1.8	1.6	5.9	

^a^Donor 2, blood condition; threefold higher platelet count, low RBC count

^b^Donor 6, under suspicion of abnormal cell content, followed up candidate

^c^Donor 9, frequent blood donor (donation of 500 mL two weeks before the test)

Previous investigations on healthy donors revealed cell findings that appeared phenotypical and morphological similar or even identical to CTCs potentially leading to increased false positive identification [24, 27, 34, 35]. A common thought was that such cell contaminations are incurred during phlebotomy and are avoided by discharging the first 5–20 mL of blood [35]. We reasoned that our diagnostic approach would be suited to address the CTC research question of non-hematopoietic cell contamination. Therefore, we separated the fresh blood sample into discharge that constitutes the first 6 mL of blood flow and into the clean sample containing 6 mL blood of the continued flow. The analysis by CD45 exclusion was limited to quantitatively investigation of the problem. According to the theory, one would expect more cells in particular larger cells in the discharge sample than in the clean blood sample and buffy coat. However, the cell counts between both samples were not significantly different (p > 0.2) counting even slightly more CD45neg cells on average in the clean blood sample (Table 5). This relationship does not change when excluding the small cell group from analysis. Our findings may be sensible in light of the blood sample resting time suggesting a negative relationship between the number of blood resting time and CD45neg cell count (r = −0.86). A significant reduction in cell counts was found for buffy coat samples (6 h, p < 0.18 and 24 h, p < 0.025).

**Fig. 7:**
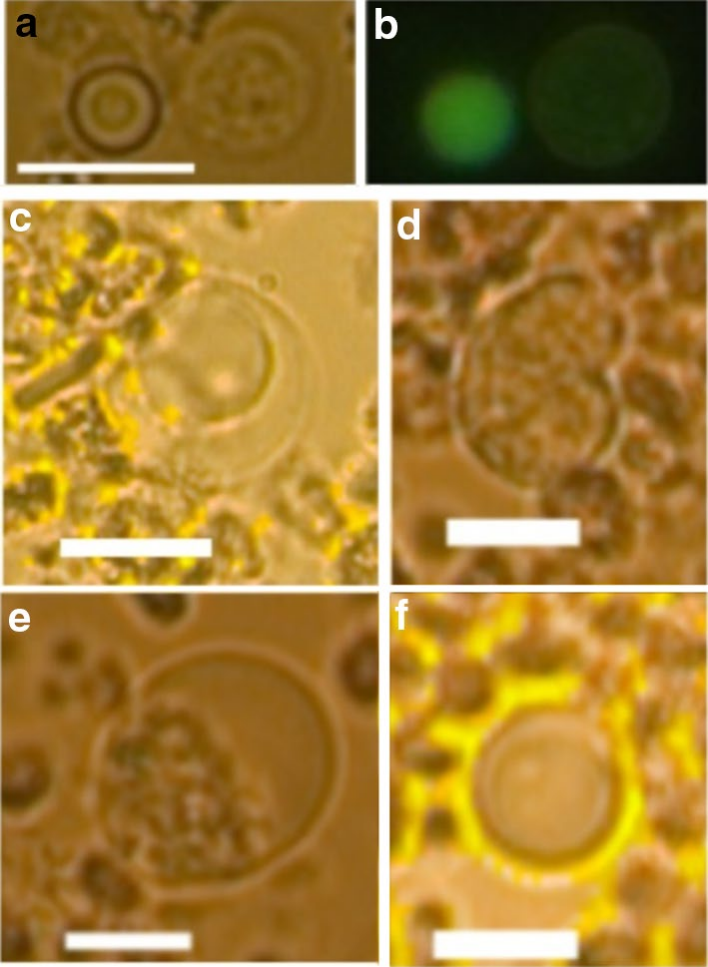
Representative images of CD45neg cells in abnormal CD45neg profiles. **a**, **b** Small CD45neg cell (*left cell*) from donor 9 under brightfield (**a**) and AO fluorescence (**b**, *left cell*). **c**–**f** Large CD45neg cells from donor 6. **c** lymphocyte-like round cell with high cytoplasm to nucleus ratio and 14 µm in diameter, **d** large red bean shaped cell with dividing nucleus measuring a horizontal diameter of 12.6 µm and vertical diameter of 20 µm, **e** largest cell with highest cytoplasm to nucleus ratio and 21 µm in diameter, **f** smallest abnormal cell measuring 10.8 µm in diameter and lowest cytoplasm to nucleus ratio. The *bars* represent a length of 10 µm

The findings of relative high quantities and a great variety of CD45neg cells encouraged us to comprehend the cell count of each donor as an individual CD45neg profile. As it is known that CD45neg cells are indicative for various health problems or conditions depending on type, quality and quantity, the idea made sense to us to qualify CD45neg cell profiles as normal and abnormal, respectively according to averaged cell quantities. In that sense, three healthy fresh blood donors showed abnormal CD45neg cell profiles. Donors 2 and 9 were in particular abnormal for small CD45neg cells that were highly similar in morphology and AO fluorescence, counting at most 16 and 7 cells, respectively (Fig. 7a, b). The average small cell count per each donor was found to measure 2.5 ± 4.2 cells (excluding donors 2 and 9, 0.9 ± 1.2 cells) per 2.5 × 10^7^ nucleated cells (Table 5). Donor 9 showed also an abnormal profile with highest total CD45neg cell count detecting at most 22 cells. The average total cell count per each donor was found to measure 5.7 ± 2.6 cells/2.5 × 10^7^ nucleated cells (Table 5). The CD45neg profile of donor 6 showed a highest level of large cells that would appear unique in morphology when compared to the CD45neg cell profiles of all other blood samples counting at most 12 such cells (Fig. 7c–f).

## Discussion

Magnetic bead-based negative selection assays have been commonly applied to isolate rare cells in particular CTCs [23]. Downstream applications such as cell analysis, cell culture or additional enrichment steps may affect the enrichment assay performance requirements leading to differences in effort of enrichment. In CTC research, optical analysis mostly employed multiple marker analysis, which allows a relative high leukocyte interference level hence, low enrichment or no enrichment at all [36]. When used in combination with molecular analysis that frequently depended on pre-amplification of nucleic acids, high enrichment may be beneficial to prevent overly false positives or unfavourable cut-off. Employing next generation sequencing methodology requires high enrichment effort as otherwise sampling becomes extremely costly. We paired our enrichment assay with simple two color fluorescence microscopy, denoted as pan-CD45neg cell isolation/detection assay following the principle of unbiased isolation and detection. Numerous nucleated cells were found to express the CD45 receptor at lowest levels (for example Fig. 5a) leading to tedious and timely microscopic reading. For that reason, leukocyte interference was sought to be kept at a practical minimum in range of 2–4 × 10^4^ nucleated cells and consequently required ultra-high, at least 625-fold depletion.

Reports about assay development and own experience let us believe that the innovation in bead based negative selection assays requires considerable attention [26, 27, 29, 37]. A quadrupole magnetic bead based cell separation system was specifically optimized for the purpose of high leukocyte depletion testing different magnetic bead systems [26]. High performance was reported for a protocol using an indirect magnetic labelling step that employs 1 µm magnetic beads and the so called tetrameric antibody complexes linker system (Stemcell Technology). An average of 3.07 log_10_ depletion and maximal 84% recovery of melanoma cell line spikes were reported. An in-house magnetic separation column based system (PowerMag) was compared with Easymag (Stemcell Technologies) and was found to be superior [27]. Indirect magnetic labelling was used choosing a cocktail of primary antibodies reactive against various leukocyte subtypes. Magnetic labelling and separation was repeated up to 4 times, reporting as high as 5.7log_10_ leukocyte depletion and a recovery of 77%. Repeated cycles of negative selection have been also applied using a microchip separation system and direct magnetic labelling with anti-CD45 reactive Dynabeads reporting 99.83% leukocyte depletion and 32% recovery of colon cancer cell line spikes [30]. While most efforts of improvement were on costs of simplicity, assay time and bead expense, we intended to include these performance criteria into the scope of optimization. Advancement in assay performance was possible by employment of the dynamic magnetic labelling procedure. The labelling method was based on the agitation of magnetic beads during incubation by slowly rotating the incubation tube in the presence of a magnetic field. A 1 min rotation cycle was found to improve capture efficiency when compared to incubation at rest, which we ascribe to increased magnetization of the target cell surface. The repetition of incubation under rotation (step 2 and 3, Fig. 1) seemed to uphold high binding reaction kinetics that would otherwise decline shortly after 1 or 2 min [33]. As a result, a relative low bead concentration and amount (bead stock estimated to contain 1.4 × 10^9^ beads per μL) produced high magnetically labelled cells within 5 min and allowed the use of a simple duopole open gradient magnetic separator requiring only 3–5 min of magnetic capture giving rise to a 10 min depletion cycle. The enrichment assay may lead as fastest high performing assay [27, 29]. Also, a simple and efficient in-house magnetic bead system was chosen using 100 nm sized beads and targeting directly the leukocyte common antigen CD45. In consequence, the use of degassed buffers, FcR blocker reagents, additional primary antibody or linker molecule incubation steps or the necessity to prepare magnetic columns was obsolete.

Similar to other investigators, repeated depletion cycles were applied to achieve ultra-high depletion [27]. However, our optimization effort went beyond the simple concept of repetition for reasons of cost effectiveness and high recovery, using different and well-adjusted bead amounts at each depletion cycle giving rise to high bead efficiency. The averaged bead efficiency of the negative selection assay measured 2.6 × 10^5^ cells/µL beads that would correspond to 30- to 40-fold depletion as determined under test conditions (Fig. 2). Therefore, the split into the depletion of high and low reacting leukocytes generated a 20-fold improvement in bead efficiency at least. The bead efficiency of the second depletion cycle measured 6.25 × 10^4^ cells/µL instead of the expected 1.4 × 10^5^ cells/µL. One reason was a sixfold increase in the debris concentration as a consequence of the incubation volume reduction from 250 to 40 µL between the two depletion cycles. Debris such as platelets and RBCs would negatively affect bead—cell binding kinetics. Also, the initial bead efficiency data arose from cell stocks comprising mostly high reacting leukocytes. It shall be noted that higher depletion rates would have been possible by employment of three or more depletion cycles however, being on costs of assay time, bead expense and recovery that we chose not to pursue. From the qualitative analysis by flow cytometry (Fig. 3), we observed selective depletion behaviour in favour for larger cell types resulting in approximately tenfold higher depletion than for what seemed to be smaller lymphocytes. Selective depletion behaviour was also reported using a similar magnetic bead system (anti-CD45, Macs beads) however, observing inefficient depletion of the granulocyte fraction [26]. It was theorized that the CD45 receptor on granulocytes may present lower affinity than lymphocytes and assumed lowered binding affinity of bead conjugated anti-CD45, so that lymphocytes would be more efficiently labelled. We believe that the cell size was an important factor in our system. Larger cells would interact comparatively more frequent with the beads than smaller cells hence, being magnetically labelled more efficiently.

As much as the requirement on enrichment or depletion factors may vary, a common directive is to minimize the loss of cells of interest. During assay evaluation experiments, highest recovery was achieved losing at most one cell when spiking 2 cells in 2 out of 3 experiments. We carried out several experiments of this kind with the conclusion that a greater cell loss had to be ascribed to the stickiness of the plastic tubes. Also, previous experimentation revealed insignificant degrees of non-specific bead-cell interaction [33]. Un-autoclaved, re-used and hand cleaned plastic tubes have been used in two out of three 8 cell spike experiments during RBC lysis and centrifugation, and is believed to be the cause of a high cell loss relative to the rest of the spiking experiments recovering only 5 out of 8 cells in both cases of mCD45 cells (Table 4). This problem was mentioned suggesting to use siliconized low-Retention micro-centrifuge tubes [38]. Further improvement may be also possible using Protein LoBind tubes (Eppendorf AG, Hamburg, Germany).

In line with high recovery, the assay was shown to enrich cells without major harm to the cells, as determined by trypan blue dye staining. The cells do not encounter any solid surfaces and shear stress during magnetic capture as it would be the case within HGMS columns. The results suggested that most harm was done by the chemical lysis of RBC, which is in accordance with previous work measuring a decrease in viability by 9–10% [29]. We measured an increase in viability after the first depletion cycle by on average 7.5% accounted for both mCD45neg cell lines, which could be attributed to cell disintegration during centrifugation, non-specific binding to the plastic tubes and to magnetic beads of in particular damaged or dead cells. The viability of the second depletion cycle showed a slightly decreased viability by on average 2% that would provide an indication of the level of untouchedness of the negative selection assay. The cell proliferation capability was somehow reduced when compared to the positive control, suggesting underlying cell damage and/or unsuited culture conditions in the presence of large amounts of platelets, unlysed RBCs and also viable nucleated CD45 positive cells (Fig. 4b). It shall be noted that the ovarian cancer cell cultures remained unaffected by microbiological contamination throughout the culture period. Comparatively little effort was required due to the simple closed system magnetic capture procedure using sterile buffers and preparing samples under laminar flow.

The technical evaluation of bead-based negative selection assays purposed for the isolation of CD45neg cells was deemed important only in a minority of published work [27]. We raised the point earlier in the discussion that the downstream application determines the performance requirements of the CD45neg cell selection assay. As a logical consequence, the current situation lacks consensus in the evaluation method of this very specific type of assay. Apparently, most applications were devoted to the detection and characterization of cancer cells. Therefore, we propose to benchmark the assay evaluation in congruence with the currently accepted reality of CTC frequencies at early stage cancer and the requirement of a low noise system. In this sense, we suggest to limit the range of L_total_ counting 2.5 × 10^7^ to 4 × 10^7^ leukocytes. Untreated blood samples should be used for cell spiking not exceeding 10 and 100 cells in recovery and cell proliferation experiments, respectively, reporting actual counts of recovered cells and proliferative activity. Cell division or outgrowth enumeration in reference to a positive control would possibly allow inter-laboratory comparison. To increase accuracy in spiking experiments and prevent false positive identification, it is certainly of advantage to mark the cells prior to the spiking experiments for later undoubted analysis and actively select the cells instead of estimating the concentration. Furthermore, we suggest employing at least two cell lines; one is related to the downstream application and another one to false positive identification.

We considered the isolation of CD45neg cells from healthy donor blood samples as a benchmark and the evaluation to be complete using the pan-CD45neg cells isolation/detection assay. Current literature reports the identification of CD45neg cells in healthy donors mostly in the specific framework of CTC detection and characterization [38–40]. For that reason, the expected average number of CD45neg cells in healthy donors remains inconclusive for our purpose. CD45neg cells with CTC-like mutations have been reported in 29.2% in healthy donor samples detecting at most 2 cells/3.2 mL PB [41]. The cell quantity is comparatively low yet comprehensible in view of using the additional FISH markers CEP8 and CEP9 beside anti-CD45 and DAPI analysis. The use of a similar marker set comprising cytokeratin (CK), CD45, DAPI and CEP8 was used in healthy donor blood detecting one CD45neg cell subtype in particular CK negative, CD45 negative, DAPI positive and CEP8 positive in 50% of the cases with a median of 2 CD45neg cells in range of 0–8 cells/3.75 mL whole blood [42]. Abnormal cell findings were reported in 93% of healthy donors based on morphological and immunologic characteristics with a mean of 5.4 cells and a range of 0–22 cells/5 mL [34]. However, numbers may not be attributed to exclusively CD45neg cells for not explicitly including CD45 cell negativity during analysis. Interestingly, this group presents a seemingly morphologically similar CD45neg cell type derived from healthy donors when compared with the type 1 cell (Fig. 6) suggesting a frequent but normal CD45neg cell type. The detection of medium-sized round EpCam neg/CD45neg cells was reported presenting a high cytoplasm to nucleus ratio from healthy donors at quantities as many as 650 cells/mL whole blood [27]. Also in this case, we may find similarities with respect to size, roundness, and cytoplasm to nucleus ratio to our finding across all sample types (Figs. 5a, 6/type 2, white arrow indications). Despite the relative scarcity and the morphological similarity, we identified this cell type as CD45 positive for presenting lowest CD45PE fluorescence. Furthermore, we may exclude the likelihood of complete CD45 down regulation caused by the enrichment treatment within given assay time as source of analytical misinterpretation. Differential expression of CD45 receptor subtypes and consequently the involvement of CD45 was reported for events such as cell activation as well as apoptosis [43, 44]. Our findings suggest greater morphological diversity and quantity of CD45neg cells in healthy donors than can be expected from current literature.

We were also interested in the problem of false positive identification in CTC diagnostics. It was theorized that venipuncture would cause contamination by all kinds of dermal cells. It has been proposed to discard a certain amount of blood as to avoid such contamination. Our results would not support this assumption and instead suggest an increasing loss of CD45neg cells with blood resting time. A similar observation of time dependent sensitivity has been reported in breast cancer experiments [45]. The results may question the common practice of initial blood flow discharge and indicate the significance to preserve blood samples.

The CD45 exclusion approach during analysis was initially intended to circumvent problems of cell marker usage such as complex multiple marker analysis, the assumption of makers and the variation in expression level [39], but most of all to obtain a complete picture of isolated CD45neg cells. Together with the findings of the high CD45neg cell diversity in healthy donors, the idea of CD45 exclusion analysis evolved into the concept of pan-CD45neg cell profiling eligible for the evaluation of the health status beyond cancer. We hypothesised that certain profiles of CD45neg cell subtypes may be indicative for various health conditions and await to be determined in the future. The findings of three donors that seemed to present abnormal profiles may support our thoughts. Donor 2 had a pre-existing blood condition of thrombocytosis (tripled platelet count compared to normal) and a relative low RBC count (determined by eye). The CD45neg cell profile showed low large and medium sized CD45neg cell counts (max. 2 cells) yet many small CD45neg cells counting at most 16 cells. It is believed that such cells would be nucleated RBCs as determined by morphology (Fig. 7a, b). The same type of cells and at elevated levels counting 7 cells was identified in the blood sample of donor 9. The blood cells of donor 9 appeared normal in regard to RBC, platelet, and leukocyte count. However, the donor was reported to be a routine blood donor having donated 500 mL 14 days before the donation for this study. Both donors were associated with slight anaemic conditions assuming the production and elevation of RBC precursors (Fig. 7a, b). Donor 9 exclusively had a profile of CD45neg cell elevation throughout all cell size groups. We can only speculate that this is result of a blood cell regeneration process. Donor 6 presented a unique set of large CD45neg cells with high as well as very low cytoplasm to nucleus ratio (Fig. 7c–f). This donor is currently followed up for being in suspicion of an underlying condition.

## Conclusion

We presented an in-house enrichment assay based on magnetic bead separation technology targeting rare CD45neg cells in peripheral blood by depleting normal cells. Technological advancements comprised simplicity, assay speed, bead expense and cell loss. We proved that highly sophisticated magnetic capture technology and lengthy cell separation procedures are avoidable by enhancements in magnetic bead labelling of target cells. Also, the simplicity of magnet capture system facilitated sterile sample preparations. Moreover, the enrichment assay was found to be highly specific yielding near complete recovery of spikes cells. As a consequence, we detected a wide range of different CD45neg cells in healthy donor peripheral blood samples at frequencies up to 22 CD45 negative cells/2.5 × 10^7^ nucleated normal cells. We addressed the research question of CD45neg cell contamination during phlebotomy concluding that such contaminations may occur yet seem to be subordinate to additional most likely blood circulating CD45 negative cells. We concluded that a short resting time of the blood sample in range of a few hours was beneficial for the isolation and detection of rare CD45 negative cells. The finding of a great variety of CD45neg cells has led us to the concept of CD45 negative profiles. Despite directing the assay purpose towards the enrichment and identification of CTCs, this concept may have potential to be extended to the liquid biopsy for screening of other malignancies that are associated with CD45 negative cells.
